# Novel intravenous human IVIG in patients with secondary immunodeficiency: Interim analysis of a multicenter, prospective, non-interventional study 

**DOI:** 10.5414/cp204975

**Published:** 2026-06-15

**Authors:** Artur Bauhofer, Silke Aigner, Stephan Borte

**Affiliations:** 1 Biotest, Dreieich, and; 2 Immune Defect Center, Clinic St. Georg, Leipzig, Germany

**Keywords:** intravenous immunoglobulin (IVIG), secondary immunodeficiency (SID), severe infections, serum IgG trough levels, non-interventional study (NIS)

## Abstract

Objective: To evaluate the effectiveness, safety, and tolerability of Yimmug, a novel, highly purified, 10% human plasma-based intravenous immunoglobulin (IVIG) preparation, in patients with secondary immunodeficiency (SID) under real-world conditions. Materials and methods: This interim analysis is based on data from a multicenter, prospective, non-interventional study conducted with out-patients in Germany. Effectiveness was assessed by changes in (i) serum IgG levels, (ii) severe infection rates, (iii) clinical symptoms, and (iv) patient-reported quality of life (QoL) at three treatment intervals (after 3, 12, and 24 IVIG infusions) compared to baseline. Safety was evaluated through documentation of adverse events (AEs), including adverse drug reactions (ADRs), while tolerability was assessed by investigators. Results: A total of 119 SID patients received 726 IVIG infusions at a median (IQR) dose of 0.3 (0.2 – 0.3) g/kg over a median (IQR) duration of 1.4 (0.4 – 2.9) years, with a median interval of 30.1 days between infusions. Serum IgG trough levels increased, with the proportion of patients achieving IgG ≥ 6 g/L rising from 30.3% at baseline to 66.7% after both 12 and 24 infusions. The mean annual rate of infections requiring antibiotics decreased from 2.3 at baseline to 0.0 after 3 and 12, and to 0.4 after 24 infusions, respectively. The new IVIG was well tolerated, and patients’ clinical symptoms and QoL improved during treatment. AEs were reported in 20 (16.8%) patients, with 41 events in total, while ADRs occurred in 9 (7.6%) patients, with 16 events. Serious AEs (SAEs) occurred in 4 (3.4%) patients, involving 5 events, but no serious ADRs (SADRs) were reported. Conclusion: This interim analysis shows that the risks are minor compared to the benefits of this novel IVIG preparation in the management of SID.

Study registration: Public registry for observational studies of the European Network of Centres for Pharmacoepidemiology and Pharmacovigilance (ENCePP), register number EUPAS41516.

**What is known about this subject**

- Secondary immunodeficiency (SID) is a common complication, notably in patients with hematological malignancies, and is associated with an elevated risk of severe and life-threatening infections.
- SID patients typically present with reduced serum immunoglobulin G (IgG) levels, a condition known as hypogammaglobulinemia.
- Current clinical guidelines recommend intravenous immunoglobulin (IVIG) therapy for SID patients with hypogammaglobulinemia who experience severe or recurrent infections.

**What this study adds**

- The data from the interim analysis support the effectiveness, safety, and tolerability of a new human plasma derived 10% IVIG preparation (Biotest, Dreieich, Germany).
- Effectiveness of the new IVIG treatment was demonstrated by multiple clinical indicators, including an increase in serum IgG trough levels, a reduction in the rate of severe infections, improvement in patients’ clinical symptoms, and enhanced patient-rated quality of life (QoL) compared to baseline.
- The new IVIG treatment was well tolerated, with a low incidence of adverse events (AEs) and adverse drug reactions (ADRs). Importantly, no serious ADRs (SADRs) were reported. These findings support a favorable relationship between the benefits and risks of this new 10% IVIG preparation in the management of SID.

## Introduction 

Secondary immunodeficiency (SID) is defined as an impairment of cell-mediated and/or antibody-mediated immunity caused by a variety of non-genetic factors, leading to an increased risk of severe and life-threatening infections [1, 2, 3]. A major cause of SID are hematological malignancies, in particular chronic lymphocytic leukemia (CLL), non-Hodgkin’s lymphoma (NHL), and multiple myeloma (MM) [1, 2, 3, 4]. The risk of developing SID in these patients is further increased by the medications used to treat them, including cytotoxic chemotherapeutics, protein kinase inhibitors, proteasome inhibitors, monoclonal antibodies, chimeric antigen receptor (CAR) T-cell therapy, and corticosteroids [1, 2, 3, 4]. Infections may account for up to 50, 33, and 22% death in CLL, NHL, and MM patients, respectively [1], emphasizing the need for accurate diagnosis and proper management protocols [4]. SID is often associated with a reduction in serum immunoglobulin concentration (hypogammaglobulinemia) and/or functionality [2, 4]. Therefore, in addition to prophylactic vaccination and antibiotic therapy, immunoglobulin replacement therapy (IgRT) is often considered to prevent severe bacterial infections, especially in SID patients with antibody deficiency [2, 4, 5]. 

Intravenous immunoglobulins (IVIG) have been used since the 1950’s, first as IgRT for the treatment of primary immunodeficiencies (PID), then additionally for the treatment of other immune-mediated and inflammatory conditions due to their anti-inflammatory and immunomodulating properties [6, 7, 8]. 

Multiple interventional and non-interventional studies support the use of IVIG to correct hypogammaglobulinemia and reduce the incidence of serious bacterial infections in patients with hematological malignancies and SID [2, 4, 9, 10, 11, 12, 13, 14, 15, 16, 17, 18, 19]. IVIG-treated patients also reported improved health status and quality of life (QoL) [14, 15, 18]. The European Medicines Agency (EMA) currently recommends IVIG administration to *“SID patients who suffer from severe or recurrent bacterial infections, ineffective antibiotic treatment and either proven specific antibody failure (PSAF) or serum IgG level of < 4 g/L”* [20]. Most other current evidence-based guidelines follow these recommendations [2, 21]. 

In clinical practice, however, the management of SID patients with hematological malignancies varies between countries, as well as regionally within countries, not always reflecting treatment guidelines [5, 8, 22, 23]. In addition, studies investigating IgRT often exhibit heterogeneous reporting (in definitions, IgG thresholds, and outcomes) [14]. These observations underscore the need for additional and robust clinical studies assessing the benefit of IgRT in these patients. 

Yimmugo (BT595; Biotest, Dreieich, Germany) is a new 10% IVIG preparation produced via a new manufacturing process intended to preserve protein integrity while reducing anti-complementary (and thus thrombogenic) activity [24]. Accordingly, this new IVIG preparation is predicted to demonstrate excellent effectiveness, safety, and tolerability. 

We present here the interim analysis of an ongoing prospective, observational, non-interventional study (NIS) aiming to assess the effectiveness, safety, and tolerability of several IVIG preparations administered to patients with diverse indications. This planned interim analysis reports the effectiveness, safety (adverse events (AEs)), and tolerability of the new IVIG preparation following 3, 12, and 24 monthly intravenous administrations in the subpopulation of SID patients (n = 119). Effectiveness of the new IVIG treatment in SID patients was evaluated via changes in (i) serum IgG trough levels, (ii) annual infection rates, (iii) clinical symptoms as per investigator’s assessment, and (iv) patient-rated QoL at the three treatment intervals compared to baseline. 

## Materials and methods 

### Study design and patients 

This prospective, observational, NIS aimed to assess the effectiveness, safety, and tolerability of IVIGs administered to patients of any age with diverse indications (including PID, SID, immune thrombocytopenia (ITP), and neuropathies). IVIG products used in the NIS were Intratect 50 mg/mL, Intratect 100 mg/mL, or the human plasma-based new IVIG 100 mg/mL (Biotest, Dreieich, Germany), prescribed at the discretion of the treating physician as routine treatment. This study also evaluated patients’ satisfaction and quality of life (QoL) over the course of IVIG treatment. Data were collected at routine clinical visits and via patient questionnaires (QoL), from patients enrolled at 63 medical centers (specialist practices and outpatient clinics) in Germany. 

Patients who received at least one IVIG infusion of studied products and signed the written informed consent were eligible for enrolment in the NIS. Patients who had participated in previous NIS using Intratect 50 mg/mL (NIS-10; ENCePP register number EUPAS7969) or Intratect 100 mg/mL (NIS-11; ENCePP register number EUPAS8040) were also eligible to enter the study. In this case, baseline data were transferred from these studies. 

The study’s primary aims were the evaluation of IVIG effectiveness and patients’ QoL, while the study secondary aims were IVIG safety and tolerability, as detailed in the Data Analysis section below. 

This study (Biotest NIS-020) was approved by the Ethics Committee of the Saxon State Medical Association (Dresden, Germany) under number EK-BR-151/20-3, dated January 31, 2023. In accordance with the current regulations of the German Medicinal Products Act (AMG), this NIS was reported to the National Association of Statutory Health Insurance Physicians (KBV), the National Association of Statutory Health Insurance Funds (GKV), the National Association of Private Health Insurance Funds (PKV), and the competent Federal Authority for approval of clinical trials involving biologicals (Paul-Ehrlich-Institut (PEI)). Accordingly, this study was conducted in compliance with Good Clinical Practice (GCP) and the Declaration of Helsinki. The study was also registered in the public registry for observational studies of the European Network of Centres for Pharmacoepidemiology and Pharmacovigilance (ENCePP) under register number EUPAS41516. 

This interim analysis evaluated the primary and secondary study aims in the subgroup of patients with SID receiving the new IVIG (100 mg/mL; Biotest). This interim analysis covered the study period from August 2, 2023 (first informed consent date) to January 2, 2025 (interim analysis cut-off date). Four time-points were considered for the interim analysis: baseline (prior to the first application of the new IVIG), after first treatment interval (3 IVIG applications, i.e., about 3 months), after fourth treatment interval (12 IVIG applications, i.e., about 12 months), and after eighth treatment interval (24 IVIG applications, i.e., about 24 months). 

### Intravenous immunoglobulin treatments 

Yimmugo (Biotest), thereafter referred to as the “new IVIG” preparation, is a human, polyvalent plasma derived immunoglobulin preparation provided as a sugar-free, glycine-stabilized, and ready-for-use solution for intravenous infusion. This new IVIG preparation contains 100 mg human plasma protein per mL (i.e., 10% solution), comprising at least 96% IgG in physiological distribution of IgG subclasses but on average 62% IgG1, 32% IgG2, 4% IgG3, and 1% IgG4. The mean (range) percentage of IgG in this new IVIG preparation was 99.9% (99.9 – 100%) of total proteins on 115 batches produced between July 2021 and May 2025 [24, 25]. Compared to the IVIG Intratect products (Biotest; [26, 27]), the new IVIG contains polysorbate 80, a lower IgA concentration (< 300 µg/mL), and presents a slightly lower pH (pH 4.4 to 5.2). The new IVIG product provides natural IgG antibodies prepared from pooled plasma obtained from several thousand blood donors. It is currently approved in Germany and some European countries in patients aged 0 years and older for the following therapeutic indications: (i) replacement therapy for PID [28] and SID (in case of severe or recurrent infections, ineffective antimicrobial treatment, proven specific antibody failure (PSAF) or serum IgG level < 4 g/L); (ii) immunomodulation for primary ITP (in case of high risk of bleeding or prior to surgery to correct the platelet count) [29], Guillain Barré syndrome, Kawasaki disease (in conjunction with acetylsalicylic acid), chronic inflammatory demyelinating polyradiculoneuropathy (CIDP), and multifocal motor neuropathy (MMN) [25]. The new IVIG product follows EMA’s guidelines on core summary of product characteristics (SmPC) for human normal immunoglobulin for intravenous administration [30]. This new IVIG is also approved in the US by the Food and Drug Administration (FDA) for the treatment of PID in patients 2 years of age or older [31]. 

In this study, prescription and dosage of the new IVIG was at the discretion of the treating physician and followed the recommendations in the manufacturer’s SmPC [25]. Each routine visit corresponded to one IVIG application (visit 1 corresponding to the first IVIG application). A treatment interval was defined as 3 IVIG applications. 

### Documented clinical parameters 

Routine clinical parameters documented by the investigators included medical history, previous medication, concomitant medication, adverse events, pre-infusion IgG levels, and risk factors associated with IVIG application. Data regarding adverse events and medical history were coded using Medical Dictionary for Regulatory Activities (MedDRA) version 27.1 [32]. Concomitant medication was coded using WHO Drug Dictionaries (version September 2024 B3 Global) following *Best Practices for the use of the WHO Drug Dictionaries’* guidelines [33, 34]. Pre-infusion IgG serum levels were measured at each visit, prior to application of the next IVIG infusion, thereby defined as “IgG trough levels”. 

### Quality of life 

The potential impact of IVIG treatment on patients’ QoL was evaluated using a visual analogue scale (VAS). Participants rated their immediate overall feeling using a VAS ranging from 1 (“very bad”) to 10 (“very good”). QoL data were collected after each 3-month treatment interval (i.e., after each 3 IVIG applications) and were analyzed at baseline and after the first, fourth, and eighth treatment interval. 

### Data analysis 

All analyses were performed using SAS (version 9.4 or higher). Only descriptive statistical methods were applied. Continuous variables were reported as the number (n) of observations and of missing data, mean, standard deviation (SD), median, and interquartile range (IQR), and, where applicable, confidence intervals. Categorical variables were reported as absolute and relative frequencies (n, %). 

Baseline data corresponded to last non-missing values documented at the beginning of the study, before the first application of the new IVIG. Baseline data from patients who had previously participated in the study Biotest NIS-010 (Intratect 50 mg/mL) or Biotest NIS-011 (Intratect 100 mg/mL;) were transferred into this study [16, 17]. 

Missing data were not replaced by imputation, with some exceptions. Incomplete dates (missing day, missing month), unless reporting AEs, were imputed as follows: missing day information was replaced by the 15^th^ of the month; missing month information was replaced by “June”. If the date of the first IVIG application was missing, it was replaced by the date of informed consent, unless the time interval between this date and the second IVIG application was > 2 months, in which case it remained as missing. In case of missing data for AE causality or seriousness, a query was sent to the reporter by Biotest. Data from subjects who terminated the study were used to the maximum extent possible. 

This interim analysis was conducted on data collected at baseline and after each 3 IVIG applications (corresponding to one treatment interval), notably after the first treatment interval (3 IVIG applications, i.e., ~3 months), after the fourth treatment interval (12 IVIG applications, i.e., ~ 12 months), and after the eighth treatment interval (24 IVIG applications, i.e., 24 months). 

Study duration was defined as the time from the date that informed consent was signed to the cut-off date of the interim analysis. Treatment duration was defined as the time between the first and the last IVIG application, adjusted to treatment breaks (defined as the sum of break days between two consecutive treatments exceeding 6 months or 182.6 days). Infusion intervals were calculated as the mean time (in months) between two infusions, disregarding treatment breaks per patient. 

Effectiveness and therapy success under real-life conditions were evaluated by analyzing (i) changes in IgG trough levels, (ii) the frequency of clinically relevant infections (i.e., antibiotics- and hospitalization-requiring), (iii) improvement in clinical symptoms as per investigator’s assessment, and (iv) QoL as per patient’s assessment. 

The annualized infection rate (AIR) was evaluated for clinically relevant infections (i.e., antibiotics-requiring infections and severe bacterial infections requiring hospitalization) and was calculated as the number of infections in the last 3 months standardized to 12 months. 

Improvement in clinical symptoms compared to the last assessment was evaluated after each treatment interval. Improvement of clinical symptoms was assessed by the respective investigators at their discretion, based on routine clinical evaluations and patient interactions, and reported according to the following 4 categories: “very good”, “good”, “moderate”, and “not satisfactory”. 

Tolerability and safety under real-life conditions were evaluated by the investigators. AEs, including suspected adverse drug reactions (ADRs), serious AEs (SAEs), and serious ADRs (SADRs), were reported. SAEs and SADRs were defined as life-threatening, resulting in death, resulting in significant disability or incapacity, requiring medical intervention or hospitalization, or being a congenital anomaly or birth defect. Infusion speed, infusion duration and infusion intervals (in weeks) were also documented. Intensity of the AEs was documented by the investigator as “mild”, “moderate”, “severe”, or “unknown”. Possible actions taken by the investigator in response to an AE (including interruption of IVIG infusion, temporary or permanent IVIG discontinuation) were reported, and AEs temporally associated with IVIG infusion (i.e., occurring during or after an infusion) were documented. AEs that occurred ≤ 72 hours after IVIG infusion were defined as infusion AEs, while those that occurred > 72 hours were not considered as infusion AEs. The number of AEs and the number of patients with AEs were reported. The percentage (%) of patients experiencing AEs was reported together with the respective 95% Clopper-Pearson confidence interval (CI) [35]. Global tolerability of the new IVIG treatment was also rated after each treatment interval per investigator’s assessment (as “very good”, “good”, “moderate”, or “not satisfactory”.), based on the frequency and severity of infusion AEs, patient-reported symptoms, and the need for dose adjustments or discontinuation. 

## Results 

### Patients’ demographics and characteristics 

**Figure 1:**
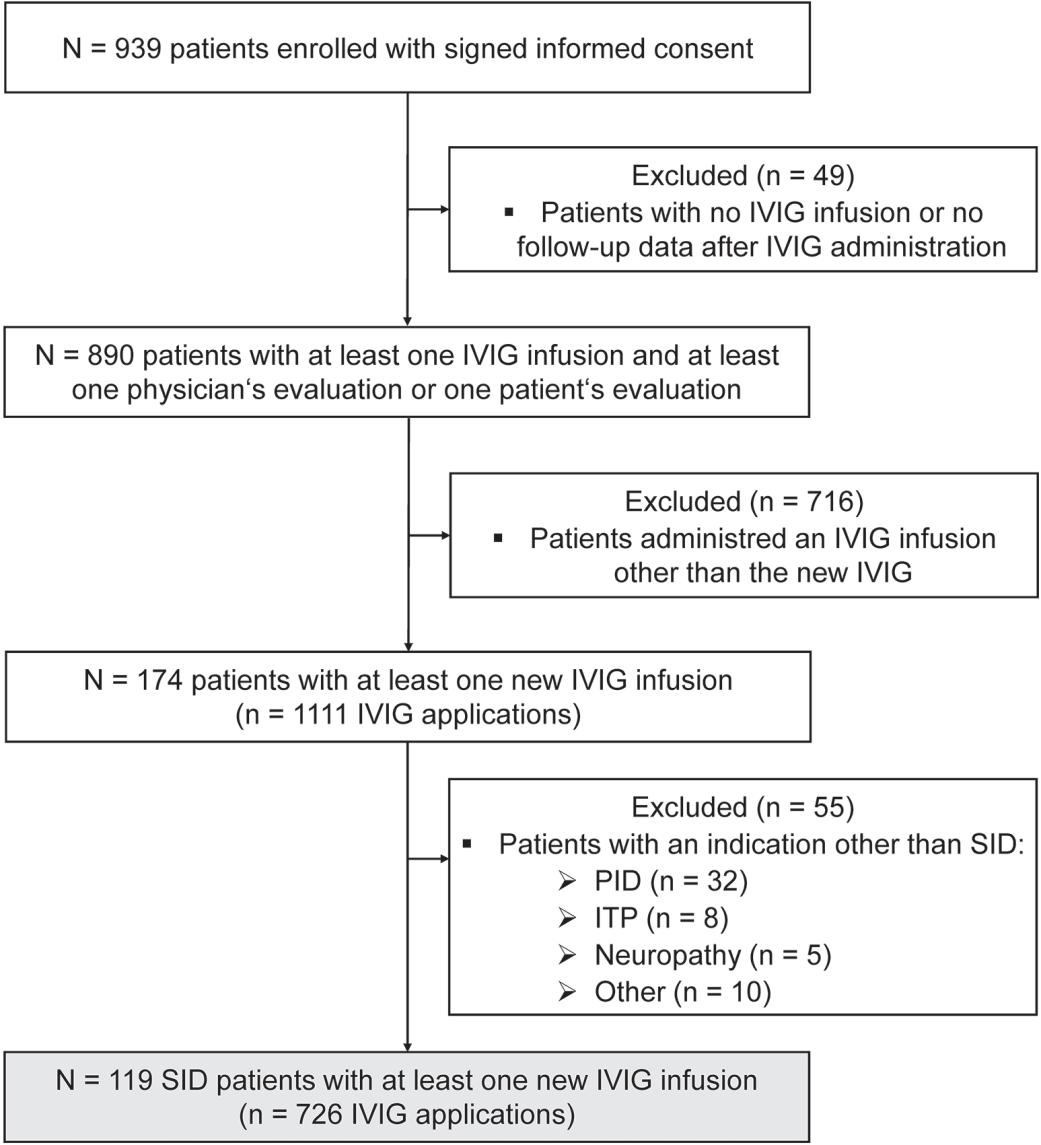
Flow diagram of interim analysis of SID patients treated with the new IVIG preparation. ITP = immune thrombocytopenia; IVIG = intravenous immunoglobulins; PID = primary immunodeficiency; SID = secondary immunodeficiency.

Of 939 patients enrolled in the study, 890 had received at least one IVIG infusion and had at least one documented physician’s or patient’s evaluation at the time of the interim analysis cut-off date. Of these 890 patients, 119 with a diagnosis of SID who had received at least one infusion of the new IVIG preparation were selected as study population for the interim analysis (Figure 1). These 119 patients were recruited at 26 medical centers in Germany. Median (IQR) study duration (defined as the time from first informed consent to cut-off date of interim analysis) was 1.9 (0.8 – 3.3) years. Early study termination occurred in 6/119 (5.0%) patients (3 deceased, 2 with AEs, and 1 not fulfilling inclusion criteria). 

Patients’ demographics and characteristics at baseline are summarized in Table 1. Age of the population ranged from 34 to 88 years with a median (IQR) of 69 (59 – 77) years. About half (51.3%) of the patients were male. None were receiving additional blood products at the time of study entry, while about two thirds (80/119 (67.2%)) had received IVIG infusions at some point prior to study entry. The most frequent underlying diseases were chronic lymphocytic leukemia (36.1%), non-Hodgkin’s lymphoma (22.7%), and multiple myeloma (21.0%). The mean (SD) AIR prior to study entry was 2.3 (3.69) for antibiotics-requiring infections and 0.7 (1.51) for severe bacterial infections requiring hospitalization (Table 1). 

### Treatment with the new 10% intravenous immunoglobulin preparation 

The median (IQR) duration of treatment of SID patients with the new IVIG preparation in this interim analysis was 1.4 (0.4 – 2.9) years (Table 2). The study population of 119 SID patients received a total of 726 IVIG applications, with a median (IQR) dose of 0.3 (0.2 – 0.3) g/kg body weight. This represented a median (IQR) annual number of infusions of 12.9 (11.5 – 15.3) and a median (IQR) time interval between infusions of 30.1 (28.2 – 34.6) days. The median (IQR) duration of an infusion was 127.6 (111.8 – 180.0) minutes, with a median (IQR) speed of 0.1 (0.1 – 0.2) g/kg/hour (Table 2). 

### Effectiveness 

Effectiveness of the new IVIG treatment in SID patients was evaluated via changes in IgG trough levels, the rate of clinically relevant infections, improvement in symptoms as per investigator’s assessment, and improvement in QoL as per patient’s rating. 

**IgG trough levels**

**Figure 2:**
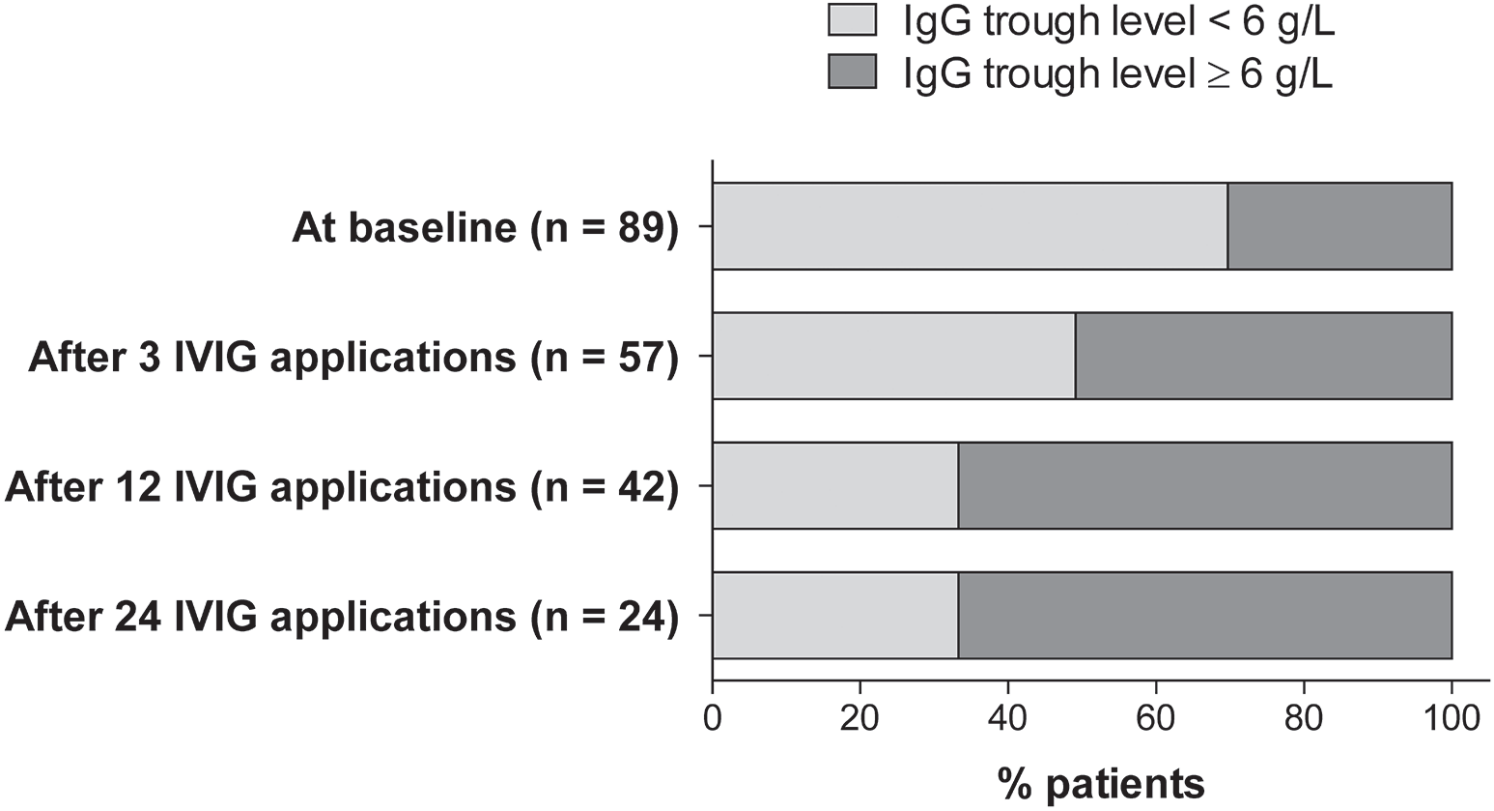
Percentage of SID patients with serum IgG trough levels < and ≥ 6 g/L at baseline and after the first, fourth, and eighth treatment interval (i.e., after 3, 12, and 24 applications of the new IVIG, respectively). The number (n) of patients with IgG trough levels considered for the respective percentage calculations is indicated on the left.

The percentage of patients with an IgG trough level ≥ 6 g/L increased from 30.3% at baseline to 50.9% after 3 IVIG applications, and 66.7% after 12 and 24 IVIG applications (Figure 2). 

Median (IQR) IgG trough levels went from 3.6 (3.1 – 3.9) g/L at baseline (n = 39) to 4.6 (3.0 – 5.9) g/L after 3 IVIG applications (n = 15), 5.4 (4.4 – 7.0) after 12 IVIG applications (n = 8), and 7.6 (4.7 – 8.5) g/L after 24 IVIG applications (n = 8). 

**Clinically relevant infections**

**Figure 3:**
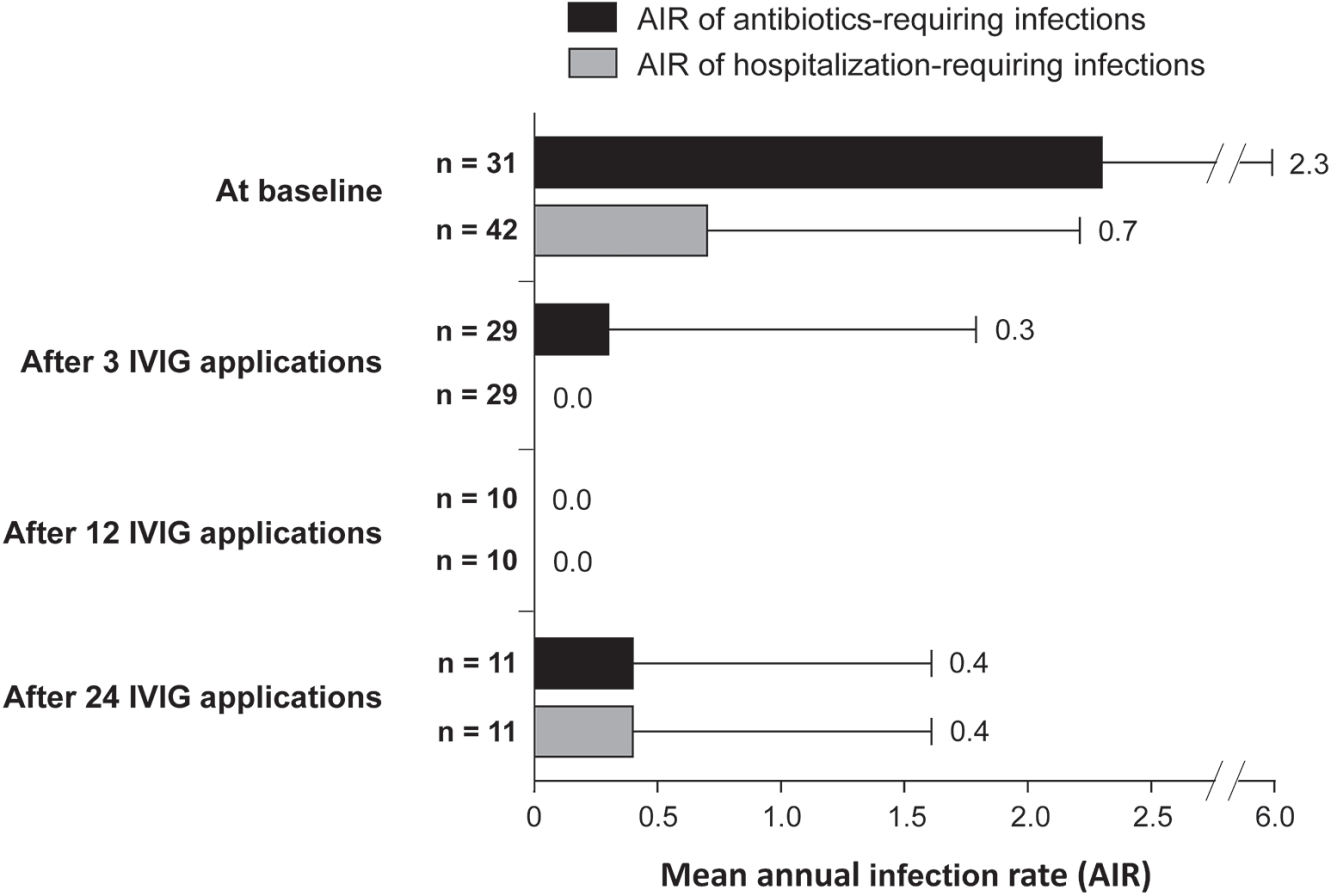
Mean and standard deviation of AIR at baseline and after the first, fourth, and eighth treatment interval (3, 12, and 24 applications of the new IVIG, respectively) in SID patients with documented infection episodes. AIR = annualized infection rate (number of infections in the last 3 months standardized to 12 months); IVIG = intravenous immunoglobulins; n = number of patients with documented infection episodes (0 to ≥ 3); SID = secondary immunodeficiency.

The mean annual infection rate (AIR) for clinically relevant infections decreased upon administration of the new IVIG preparation. The mean AIR for antibiotics-requiring infections went from 2.3 infections per patient-year at baseline (Table 1) to 0.0 – 0.4 after 3 – 24 IVIG applications (Figure 3). Similarly, the mean AIR for severe bacterial infections requiring hospitalization went from 0.7 infection per patient-year at baseline (Table 1) to 0.0 – 0.4 after 3 – 24 IVIG applications (Figure 3). 

**Health status improvement**

Most patients were assessed with good to very good improvement in their clinical symptoms by the investigator after the first (24/27 (88.9%)), fourth (10/10 (100%)), and eighth (9/10 (90.0%)) treatment intervals (Table 3). 

**Quality of life**

As QoL measurement, SID patients receiving the new IVIG preparation rated their immediate overall feeling using a visual analogue scale (VAS), scored from 1 (“very bad”) to 10 (“very good”). QoL data were available at baseline and after 3 and 12 IVIG applications. QoL improved following administration of the new IVIG, with a mean VAS score of 5.8 at baseline increasing to 6.4 after 3 and 12 IVIG applications (Table 4). 

### Safety and tolerability 

**Safety**

Out of the 119 patients of the study population, 20 (16.8%) experienced a total of 41 AEs, of which 5 were SAEs and affected 4/119 (3.4%) patients (Table 5). Most AEs (35/41 (85.4%)) were rated as mild or moderate in 17/119 (14.3%) patients. Of the 20 patients with AEs, 9 suffered from 16 ADRs (defined as related to IVIG infusion), none of which were rated as SADRs (0/41 (0.0%)) (Table 5). Most AEs (35/41 (85.4%)) led to recovery or were recovering at the time of documentation, and 2/41 (4.9%) led to death (Table 5). 

With a total of 726 IVIG infusions, the overall incidence of AEs and ADRs was estimated at 0.056 (41/726) and 0.022 (16/726) per infusion, respectively, in the study population. 

Most (22/41 (53.7%)) AEs occurred > 72 hours after IVIG infusion (hence, not considered as infusion-related), while 14/41 (34.1%) occurred during or < 2 hours after infusion and 5/41 (12.2%) between 2 and 72 hours (defined as infusion AEs). Of the 41 AEs, 15 (36.6%) resulted in interrupting infusion or temporally discontinuing IVIG application, while none (0/41 (0.0%)) led to the permanent discontinuation of IVIG treatment. 

The 41 reported AEs are further described in Supplemental Table S1, the 16 ADRs in Supplemental Table S2, and the 5 SAEs in Supplemental Table S3. The most frequent AEs were mild to moderate infections, affecting 11 (9.2%) patients (one infection episode per patient) (Supplemental Table S1). Only 2/119 (1.7%) patients experienced an AE of moderate intensity related to a known IVIG risk: a hypersensitivity event for 1 patient (also rated as ADR (Supplemental Table S2)) and an interference of IVIG with serological testing (positive candida test) for the other. None of the reported AEs were related to the other known IVIG-associated risks such as thromboembolism, acute renal failure, aseptic meningitis, hemolytic anemia, transfusion-related acute lung injury (TRALI), leukopenia or neutropenia, transmission of infective agents via blood product, and interference of IVIG with live attenuated virus vaccines. Regarding pain, infusion ADRs (< 72 hours after infusion) were documented for only one patient (0.8%) each with moderate headache or moderate back pain (Supplemental Table S2). The 5 SAEs experienced by 4 patients were disease progression, multiple organ dysfunction syndrome, gastric stenosis, gastritis, and abdominal operation (Supplemental Table S3). 

**Tolerability**

The tolerability of the new IVIG treatment was evaluated by the investigator, based on safety data and patient-reported symptoms. Most patients were rated with good to very good tolerability after the first (27/28 (96.4%)), fourth (9/10 (90.0%)), and eighth (9/10 (90.0%)) treatment intervals (Table 6). 

## Discussion 

This interim analysis investigated the effectiveness, tolerability and safety of a new generation IVIG preparation in patients with SID under real-world conditions. 

This analysis, conducted in a subpopulation of 119 patients, revealed good effectiveness and a favorable tolerability and safety profile for this new IVIG preparation. The proportion of patients with serum IgG trough levels ≥ 6 g/L increased from 30.3 to 66.7% upon IVIG treatment, confirming the correction of hypogammaglobulinemia by IgRT shown in previous studies [15, 16, 18, 23, 36]. Concomitantly, the annual infection rate for clinically relevant infections decreased (mean AIR ≤ 0.4), while patients’ clinical symptoms and QoL improved. This concurs with multiple studies demonstrating a reduced incidence of infection following IVIG treatment [2, 4, 9, 10, 11, 12, 13, 14, 15, 16, 17, 18, 19, 23, 36, 37] and improved health status and QoL [14, 15, 16, 17, 18]. It should be noted that the beneficial impact of the new IVIG treatment on reducing infection rates was observed using the median (IQR) dosage of 0.3 (0.2 – 0.3) g/kg and was hence in line with EMA’s recommendation of applying between 0.2 and 0.4 g/kg IVIG in SID patients [20, 30]. 

The new IVIG was well tolerated. No SADR and no known class risks associated with IVIG administration (including thromboembolism, acute renal failure, aseptic meningitis, hemolytic anemia, TRALI, leukopenia, or neutropenia) were observed. The safety profile of the new IVIG was favorable, with 9/119 (7.6%) patients with ADR, 20/119 (16.8%) patients with AEs, of which 4 (3.4%) experienced SAEs. The proportion of the new IVIG-treated patients experiencing AEs and ADRs in this interim analysis was low and within the range reported for most other IVIG products [16, 17, 23, 36, 38, 39, 40, 41]. Notably, no SADRs were documented, aligning with findings from large-cohort IVIG studies that report a low incidence of SADRs in patients with SID [39, 40]. In our small-cohort interim analysis, one ADR was classified as moderate hypersensitivity, and no thromboembolic events were observed. These findings are consistent with previously reported low incidences of hypersensitivity (0.3%) and thromboembolism (0.1%) in a large cohort of 2,397 patients [40]. The favorable safety profile observed in this interim analysis warrants confirmation through final analysis of the complete study cohort. 

Altogether, these interim data demonstrated a positive benefit-risk profile for the new IVIG preparation in SID patients. No unexpected events were observed, and the safety profile of the new IVIG was in line with the current label for IVIG [20, 30]. As expected from a population of SID patients with hematological malignancies, most patients were elderly, with a median (IQR) age of 69 (59 – 77) years. Accordingly, our findings may be considered representative for adults and elderly patients. 

Our study presents several notable strengths, including its multicenter design, its execution in a real-world clinical setting, and the inclusion of a large SID subpopulation. This subpopulation is well-represented by the primary hematological malignancies associated with SID, namely chronic lymphocytic leukemia, non-Hodgkin’s lymphoma, and multiple myeloma [1, 2, 3, 4], thereby enhancing the generalizability and relevance of our findings. 

Our study has several limitations. First, its observational, non-interventional design inherently restricts causal inference. Second, as an interim analysis, the findings are limited to descriptive statistics. Additionally, approximately two-thirds of the enrolled patients had received prior treatment with other IVIG products before study entry, introducing heterogeneity into the study population. Furthermore, some data – such as patient-reported QoL – were incomplete at the interim analysis cut-off date. The final analysis is expected to provide a more comprehensive and robust evaluation of the new IVIG’s effectiveness, tolerability, and safety, not only in patients with SID but also in those with other clinically relevant indications, including PID, ITP, and various neuropathies (e.g., chronic inflammatory demyelinating polyneuropathy, multifocal motor neuropathy, Guillain-Barré syndrome, and myasthenia gravis). 

## Conclusion 

Our interim analysis suggests that this novel, highly purified, 10% IVIG preparation –developed using a novel manufacturing process designed to preserve protein integrity and minimize thrombogenic potential [24] – demonstrates a favorable safety profile and holds promise for therapeutic efficacy in SID patients. Confirmation of these encouraging interim findings through final study analysis is warranted, along with exploration of their applicability to other clinically relevant indications. 

## Acknowledgment 

The authors thank Anne Rascle of AR Medical Writing (Regensburg, Germany) for providing medical writing support, which was funded by Biotest (Dreieich, Germany), in accordance with Good Publication Practice (GPP 2022) guidelines (https://www.ismpp.org/gpp-2022; accessed on December 5, 2025). 

## Authors’ contributions 

A. Bauhofer: study design and supervision, data analysis and interpretation, main author of the publication. S. Aigner: data analysis and interpretation. S. Borte: study result interpretation. 

## Funding 

The study was funded by Biotest (Dreieich, Germany). 

## Conflict of interest 

Artur Bauhofer and Silke Aigner are employees of Biotest. 

**Table 1.** Patients’ demographics and characteristics at baseline.

Characteristics	SID patients receiving the new IVIG (N = 119)
Age in years, median (IQR)	69.0 (59.0 – 77.0)
Age in years, range	34.0 – 88.0
Body weight in kg, median (IQR)	75.5 (66.0 – 87.5)
Sex, N (%)	
Male	61 (51.3%)
Female	58 (48.7%)
Underlying diseases, N (%)	
Chronic lymphocytic leukemia (CLL)	43 (36.1%)
Non-Hodgkin’s lymphoma (NHL)	27 (22.7%)
Multiple myeloma (MM)	25 (21.0%)
Breast cancer	1 (0.8%)
Prostate cancer	1 (0.8%)
Other cancers	15 (12.6%)
Non-cancerous disease	6 (5.0%)
Missing information	1 (0.8%)
Cancer treatment in the last 2 weeks prior to study entry, N (%)^1^	
Yes	20 (16.8%)
No	27 (22.7%)
Type of cancer treatment in the last 2 weeks prior to study entry, N (%)^2^	
Chemotherapy	14 (11.8%)
Surgery	3 (2.5%)
Monoclonal antibodies	2 (1.7%)
Radiotherapy	1 (0.8%)
Treatment with another IVIG prior to study start	80 (67.2%)
Yes	80 (67.2%)
No	39 (32.8%)
Infection episodes in the last 3 months prior to study start	
AIR of antibiotics-requiring infections (n = 31), mean (SD)^3^	2.3 (3.69)
AIR of hospitalization-requiring infections (n = 42), mean (SD)^3^	0.7 (1.51)

^1^Cancer treatment only documented for 47/119 (39.5%) patients. ^2^For the patients with documented cancer treatment (n = 20). ^3^Excluding patients with missing data; expressed as infections per patient-year. AIR = annualized infection rate (number of infections standardized to 12 months); IQR = interquartile range; IVIG = intravenous immunoglobulins; N = number of patients per category; n = number of patients with documented infection episodes (0 to ≥ 3); SD = standard deviation; SID = secondary immunodeficiency.

**Table 2.** Dosing, number, and frequency of applications of the new IVIG.

New IVIG treatment characteristics	SID patients receiving the new IVIG (N = 119)
Observation period in years, median (IQR)^1^	1.9 (0.8 – 3.3)
Treatment duration in years, median (IQR)^2^	1.4 (0.4 – 2.9)
Total number of applications, N	726
Annual number of infusions, median (IQR)	12.9 (11.5 – 15.3)
Time interval between infusions in days, median (IQR)	30.1 (28.2 – 34.6)
Dose in g/kg body weight, median (IQR)	0.3 (0.2 – 0.3)
Infusion duration in minutes, median (IQR)	127.6 (111.8 – 180.0)
Infusion speed in g/kg body weight/hour, median (IQR)	0.1 (0.1 – 0.2)

^1^Study duration, defined as the time from first informed consent to cut-off date of interim analysis. ^2^Time between the first and last IVIG application. IQR = interquartile range; IVIG = intravenous immunoglobulins; N = number of patients; SID = secondary immunodeficiency.

**Table 3.** Improvement in clinical symptoms since the last assessment, evaluated by the investigator after the first, fourth, and eighth treatment interval (3, 12, and 24 applications of the new IVIG, respectively) in SID patients.

Improvement of clinical symptoms	After 3 IVIG applications (n = 27)*	After 12 IVIG applications (n = 10)*	After 24 IVIG applications (n = 10)*
Very good, N (%)	4 (14.8%)	3 (30.0%)	1 (10.0%)
Good, N (%)	20 (74.1%)	7 (70.0%)	8 (80.0%)
Moderate, N (%)	3 (11.1%)	0 (0.0%)	1 (10.0%)
Not satisfactory, N (%)	0 (0.0%)	0 (0.0%)	0 (0.0%)

*Excluding patients with missing data. IVIG = intravenous immunoglobulins; n = number of patients with documented assessment of clinical symptom improvement; N (%) = number and percentage of patients in each category.

**Table 4.** Quality of life of SID patients self-evaluated by visual analogue scale at baseline and after the first, fourth, and eighth treatment interval (3, 12, and 24 IVIG applications, respectively).

Quality of life	At baseline (n = 28)*	After 3 IVIG applications (n = 38)*	After 12 IVIG applications (n = 36)*	After 24 IVIG applications (n = 0)*
VAS score, mean (SD)	5.8 (1.77)	6.4 (1.45)	6.4 (2.03)	No data

*Excluding patients with missing data. IVIG = intravenous immunoglobulins; SD = standard deviation; VAS = visual analogue scale.

**Table 5.** Adverse events in the population of SID patients receiving the new IVIG (N = 119).

All events	Number of patients (%) with at least one event [95% CI]^1^	Number of events
Adverse events	20 (16.8%) [10.6 – 24.8%]	41
Adverse event severity		
Mild	9 (7.6%) [3.5 – 13.9%]	14
Moderate	8 (6.7%) [2.9 – 12.8%]	21
Severe	4 (3.4%) [0.9 – 8.4%]	5
Unknown	1 (0.8%) [0.0 – 4.6%]	1
Adverse drug reaction	9 (7.6%) [3.5 – 13.9%]	16
Serious adverse events	4 (3.4%) [0.9 – 8.4%]	5
Serious adverse drug reactions	0 (0.0%) [0.0 – 3.1%]	0
Fatal adverse event	2 (1.7%) [0.2 – 5.9%]	2

^1^95% Clopper-Pearson confidence interval (CI) applied to the percentage of patients. CI = confidence interval; IVIG = intravenous immunoglobulins; SID = secondary immunodeficiency.

**Table 6.** Tolerability of the new IVIG preparation after the first, fourth, and eighth treatment interval (3, 12, and 24 IVIG applications, respectively) in SID patients, rated per investigator’s assessment.

Tolerability of the new IVIG infusion	After 3 IVIG applications (n = 28)*	After 12 IVIG applications (n = 10)*	After 24 IVIG applications (n = 10)*
Very good, N (%)	7 (25.0%)	3 (30.0%)	6 (60.0%)
Good, N (%)	20 (71.4%)	6 (60.0%)	3 (30.0%)
Moderate, N (%)	1 (3.6%)	1 (10.0%)	1 (10.0%)
Not satisfactory, N (%)	0 (0.0%)	0 (0.0%)	0 (0.0%)

*Excluding patients with missing data. IVIG = intravenous immunoglobulins; n = number of patients with rated tolerability; N (%) = number and percentage of patients in each category; SID = secondary immunodeficiency.

### Supplemental material

Supplemental material Tables
