# Supplementary material for: Novel intravenous human IVIG in patients with secondary immunodeficiency: Interim analysis of a multicenter, prospective, non-interventional study
Source: Int J Clin Pharmacol Ther. 2026 Jun 15;64(8):416–27. doi: 10.5414/cp204975 (PMC13409343; doi:10.5414/cp204975)
Supplement: Supplemental material [file intjclinpharmacol-64-416-S01.pdf]

Supplementary Tables

Table S1. Description of all 41 AEs observed in 20 SID patients receiving the new IVIG, according to MedDRA System Organ Class (SOC) and Preferred Terms (PT) classification.

MedDRA SOCs, Preferred Terms (intensity)	Number of patients (%)	Number of events
Infections and infestations, including nasopharyngitis, pneumonia, and respiratory tract infection (mild to moderate)	11 (9.2%)	11
General disorders and administration site conditions, such as chills, fatigue, pyrexia, disease progression, and multiple organ dysfunction syndrome (mild to severe)	7 (5.9%)	8
Gastrointestinal disorders, specifically gastric stenosis, gastritis, and nausea (moderate to severe)	4 (3.4%)	4
Injury, poisoning and procedural complications, specifically infusion-related reaction (mild to moderate)	3 (2.5%)	3
Respiratory, thoracic and mediastinal disorders, specifically cough and dyspnoea (mild to moderate)	3 (2.5%)	3
Vascular disorders, specifically hypertension (mild to moderate)	2 (1.7%)	3
Nervous system disorders, specifically headache and polyneuropathy (mild to moderate)	2 (1.7%)	2
Investigations, specifically candida test positive and oxygen saturation decreased (moderate)	1 (0.8%)	2
Skin and subcutaneous tissue disorders, specifically erythema (moderate)	1 (0.8%)	1
Immune system disorders, specifically hypersensitivity (moderate)	1 (0.8%)	1
Musculoskeletal and connective tissue disorders, specifically back pain (moderate)	1 (0.8%)	1
Renal and urinary disorders, specifically polyuria (moderate)	1 (0.8%)	1
Surgical and medical procedures, specifically abdominal operation (severe)	1 (0.8%)	1

Abbreviations: AEs, adverse events; MedDRA, medical dictionary for regulatory activities; SID, secondary immunodeficiency; SOC, System Organ Class.

Table S2. Description of 16 ADRs observed in 9 SID patients receiving the new IVIG, according to MedDRA System Organ Class (SOC) and Preferred Terms (PT) classification.

MedDRA SOCs, Preferred Terms (intensity)	Number of patients (%)	Number of events
Injury, poisoning and procedural complications, specifically infusion-related reaction (mild to moderate)	3 (2.5%)	3
Vascular disorders, specifically hypertension (mild to moderate)	2 (1.7%)	3
Gastrointestinal disorders, specifically nausea (moderate)	2 (1.7%)	2
General disorders and administration site conditions, specifically chills and malaise (moderate)	2 (1.7%)	2
Nervous system disorders, specifically headache (moderate)	1 (0.8%)	1
Skin and subcutaneous tissue disorders, specifically erythema (moderate)	1 (0.8%)	1
Immune system disorders, specifically hypersensitivity (moderate)	1 (0.8%)	1
Infections and infestations, specifically cystitis (moderate)	1 (0.8%)	1
Musculoskeletal and connective tissue disorders, specifically back pain (moderate)	1 (0.8%)	1
Respiratory, thoracic and mediastinal disorders, specifically dyspnoea (moderate)	1 (0.8%)	1

Abbreviations: ADRs, adverse drug reactions; MedDRA, medical dictionary for regulatory activities; SID, secondary immunodeficiency; SOC, System Organ Class.

Table S3. Description of the 5 SAEs observed in 4 SID patients receiving the new IVIG, according to MedDRA System Organ Class (SOC) and Preferred Terms (PT) classification.

MedDRA SOCs, Preferred Terms (intensity)	Number of patients (%)	Number of events
General disorders and administration site conditions, specifically disease progression and multiple organ dysfunction syndrome (severe)	2 (1.7%)	2
Gastrointestinal disorders, specifically gastric stenosis and gastritis (severe)	2 (1.7%)	2
Surgical and medical procedures, specifically abdominal operation (severe)	1 (0.8%)	1

Abbreviations: MedDRA, medical dictionary for regulatory activities; SAEs, Severe adverse events; SID, secondary immunodeficiency; SOC, System Organ Class.
